# Psychological and Social Predictors of Poverty: Differences Between Lesbian and Bisexual Women

**DOI:** 10.1016/j.whi.2024.10.004

**Published:** 2024-12-04

**Authors:** Bianca D.M. Wilson, Andy Lin, Lauren J.A. Bouton

**Affiliations:** a Department of Social Welfare, UCLA, Los Angeles, California; b Office of Advanced Research Computing, UCLA, Los Angeles, California; c Williams Institute, UCLA, Los Angeles, California

## Abstract

**Purpose:**

Research has demonstrated that sexual minority populations are more likely to experience poverty than sexual majority populations and that many of these disparities are driven by specific sexual minority subgroups, including cisgender bisexual women. Yet, little is known about the factors associated with economic insecurity that explain the intragroup differences in economic outcomes among sexual minorities, particularly among those of the same gender (i.e., cisgender bisexual vs. lesbian women).

**Methods:**

We used a U.S. national probability sample of non-transgender sexual minority adults to assess the relationship between poverty and demographic (age, race/ethnicity, education), psychological (psychological distress, self-acceptance, felt stigma, and experienced discrimination), and social (outness, partnership and parental status, partner gender, and gender expression) characteristics for each subgroup of women, lesbian/gay (n = 324) and bisexual (n = 355). We calculated odds ratios and adjusted odds ratios (AORs) estimated from logistic regression models that relate risk factors to poverty.

**Results:**

Race/ethnicity (i.e., identifying as Black) and education (i.e., having a high school diploma or less) were associated with living in poverty for both groups. The role of minority stressors, such as outness, everyday discrimination, and internalized homophobia did not strongly predict poverty for either group. However, reports of experienced stigma related to one’s sexual orientation and masculine gender expression were associated with poverty among lesbians but not for bisexual women, and having children was a strong predictor of poverty for bisexual women but not lesbians.

**Conclusions:**

These findings suggest that policy, advocacy, and service interventions should consider tailoring approaches to address poverty for bisexual and lesbian women differently.

Economic instability remains a consistent risk factor and driver of health inequities in the United States (Adler & Ostrove, 1999; Beech et al., 2021; Fogel, 2004; Sapolsky, 2005). Poverty, a major indicator of economic instability, is one of the domains in which there are economic disparities between sexual minority and majority populations (Baams et al., 2019; Badgett et al., 2019; Russomanno & Jabson Tree, 2020; Singh et al., 2022; Wilson, Choi, et al., 2020). Poverty is officially defined as living below an income threshold set by the U.S. government in relationship to the number of people in the household (U.S. Census Bureau, 2023). For example, an adult and two children with an annual gross household income of less than $23,578 would fall under the definition of living in poverty in 2022. Research has demonstrated for decades that sexual minority populations, defined by sexual identities (lesbian, gay, bisexual) or relationship composition (i.e., same-sex couples), are more likely to experience poverty than heterosexual people or “opposite-sex” couples (Badgett, 2018; Badgett & Schneebaum, 2015; Schneebaum & Badgett, 2019). However, recent research has highlighted that many of the economic differences identified between sexual minority and majority populations are driven by specific sexual minority subgroups, including cisgender bisexual women (Badgett et al., 2019). While prevailing theories of the impact of sexual minority stigma may explain multiple disparate outcomes among lesbian, gay, and bisexual (LGB) people as a whole, little is known about the factors associated with economic insecurity that explain the intragroup economic differences among sexual minorities. In the current study, we address these gaps by examining the relationship between psychological and household characteristics and poverty status, comparing the strength of predictors between cisgender bisexual and lesbian women.

## Poverty and Sexual Orientation Identity

Over a decade of empirical research on sexual orientation identity differences in economic instability has demonstrated the problems with the myth of the “white affluent male” as the defining demographic of the lesbian, gay, bisexual, and transgender (LGBT) population (Badgett, 1995, 2018). Starting in 2011, census data that included the gender of co-habitating adults allowed for studies of household income among same-sex couples. Research showed that same-sex couples of women had significantly lower incomes than different-sex couples and same-sex couples of men (Badgett et al., 2013; Gates, 2013). This was an important movement in sexual orientation economic status research as it confronted the mythology about LGBT communities. Yet, it was limited by its defining of sexual minorities by who they were partnered with in one moment of time. Particularly among plurisexual identified people, such as bisexual people, information on partner gender in a one-time cross-sectional survey does not indicate sexual identity. As more population-based surveys began including measures of sexual orientation identity (e.g., heterosexual/straight, lesbian, bisexual), research examining the relationship between sexual minority identities and income progressed.

## Economic Disparities Among Sexual Minority Women

More nuanced research on economic insecurity among sexual minorities has identified cisgender bisexual women as a group with high proportions living with low incomes, compared with all other sexual minority cisgender groups and compared with cisgender straight men and women (Badgett, 2018; Badgett et al., 2019). Though poverty rates have fluctuated over the past 5 years (Creamer et al., 2022; Shrider & Creamer, 2023), partly a function of the COVID-19 pandemic and federal relief administered in response, patterns of LGBT economic disparities have remained consistent. In 2019, 23% of LGBT adults were living with incomes below the federal poverty level, a significantly higher rate compared with 16% of cisgender straight adults (Wilson et al., 2023). Further, more cisgender bisexual women than any other sexual orientation group were living in poverty: 30%, compared with 14% among cisgender lesbian women and 18% among cisgender straight women (Wilson et al., 2023).

Researchers, service providers, and advocates have struggled to explain why these subgroup differences in poverty occur. When the focus is on comparisons between sexual minority and majority populations, efforts to explain LGB disparities typically rely on theories that map out differences in experiences of discrimination due to sexual minority status. For example, the minority stress framework is an evidence-based theory explaining several dimensions of health disparities among sexual minority people compared with people who are heterosexual (Frost et al., 2015; Institute of Medicine, 2011; Lick et al., 2013; Meyer, 2003). In applying this framework to economic well-being, differences in employment and economic outcomes are understood as a function of additional stress burden, and thus decreased mental health, experienced by sexual minorities who must cope with anti-LGBT bias (see, e.g., Gorman et al., 2015; McGarrity, 2014; Ross et al., 2016). Similarly, scholars have identified forms of sexual orientation–related discrimination that may directly lead to lower incomes, such as denials of hiring or promotions because of anti-LGBT bias (Badgett et al., 2021; Sears et al., 2021). Differences between sexual minority men and women’s incomes and rates of poverty appear adequately explained by persistent findings that men are paid more, on average, than women in the United States (Schneebaum & Badgett, 2019). However, it is less clear how to explain differences among sexual minority women subpopulations, such as cisgender bisexual women compared with cisgender lesbian women, who theoretically might both experience sexual orientation–related discrimination and gender-based pay equity concerns. There are several domains of psychological and social characteristics in which bisexual and lesbian-identified cisgender women are known to differ, and these may help to explain differences in economic status between these two groups.

## Psychological and Social Characteristics Across Sexual Orientation

Research on economic concerns among cisgender bisexual women has suggested that bisexual-specific minority stressors, such as biphobia or anti-bisexual bias, may explain the higher rates of poverty in this group (Bostwick & Hequemborg, 2014). Research on bisexual women’s experiences more generally has demonstrated the prevalence and negative impacts of antibisexuality bias within the dominant heterosexual U.S. culture and within LGBT community spaces (Friedman et al., 2014; Mulick & Wright, 2011). Qualitative research on the experiences of LGBTQ people living with low incomes found that biphobia and anti-bisexual bias were salient aspects of cisgender bisexual women’s experiences, particularly when family members and cisgender male partners were the ones exhibiting the biphobia and bias (Wilson, Gomez, et al., 2020). However, cisgender bisexual women participants in that study did not discuss sexual orientation–related discrimination in many of the settings that may have direct material impacts on economic status, such as employment and housing. Among lesbians in that study, key factors discussed in relationship to their challenges with work and employment services included discrimination based on gender expression (i.e., appearing gender nonconforming, masculine, or identifying as “butch” or “stud”) and managing children and family needs (Wilson, Gomez, et al., 2020).

It is possible that psychological distress may also play a role in explaining economic outcome disparities for bisexual women. Bisexual women report higher levels of depression and psychological distress compared with other sexual minority subgroups and compared with all heterosexual people (Bostwick et al., 2010; Conron et al., 2010; Ross et al., 2018; Wilson et al., 2021). Mental health disorders are known predictors, as well as outcomes, of financial strain (Hudson, 2005; Sareen et al., 2011; Yu & Williams, 1999). In addition to biphobia, research has demonstrated an association between other related minority stressors and mental health among bisexual adults, such as connectedness to the LGBT community (Kertzner et al., 2009; Lamb et al., 2017), self-acceptance or degree of internalized biphobia (Camp et al., 2020), and level of sexual identity disclosure (i.e., outness) (Feinstein et al., 2019). These may be important mechanisms to understand the relationship between mental health and poverty among bisexual women. However, lesbians also experience minority stressors. Minority stressors and sexual minority experiences among lesbian-identified women (i.e., gender nonconformity, anti-gay discrimination and violence, identity concealment, internalized homophobia) are also associated with mental health concerns, such as lifetime mood and anxiety disorders (Bostwick et al., 2010; Hernandez et al., 2024; Meyer, 2003).

It is possible that some of the economic differences between bisexual and lesbian women are a function of differences in social or contextual experiences between these LGB subgroups. For example, the U.S. population of lesbian women is older and has higher levels of formal education, on average, than bisexual women (Jones, 2024; Wilson et al., 2021). Lesbian women are also less likely to have young children in the home than bisexual women (Wilson & Bouton, 2024). Being younger, having lower levels of education, and parenting are associated with poverty and economic strain in the general population and among LGBT people, specifically (Badgett et al., 2019; Bleiweis et al., 2020; Wilson et al., 2023). Yet, lesbians, particularly those from racialized groups, are more likely to have children, indicating that parenting is likely a relevant factor for this subgroup. Another social factor could be the gender of romantic partners. An over-whelming majority of bisexual women who are partnered report being partnered with cisgender men, most of whom are heterosexual (Wilson et al., 2022). In addition to the potential negative impact on mental health that gender and sexual orientation discordant coupling (e.g., a bisexual woman partnered with a heterosexual man) can have for bisexual women (Mark et al., 2020; Vencill et al., 2018; Wilson et al., 2022), there may be economic implications. Moreover, cisgender men tend to make more money than cisgender women overall, and therefore being partnered with a cisgender man may provide a buffer from economic instability for bisexual women compared with lesbian women. Yet, it is not clear whether partner status, partner characteristics, or parenting status are factors for experiencing low incomes or poverty among these subgroups of sexual minority women.

Taken together, prior research indicates that both lesbian and bisexual women likely experience a range of factors that explain higher rates of poverty compared with straight and sexual minority men (Badgett, 2018; Wilson, Gomez, et al., 2020). Research also seems to highlight some areas in which differences in psychological and social characteristics between lesbian and bisexual women might explain higher rates of poverty among bisexual women (Badgett et al., 2019; Fredriksen-Goldsen et al., 2010). Given the constellation of factors that may be associated with economic status among both lesbian and bisexual women, the aim of the current study was to explore which factors predict poverty for each subgroup.

## Intersectionality, Health, and Economic Well-being

Although this study is most focused on understanding the variability in predictors of poverty among sexual minority women, it is guided by the collection of frameworks that take into account the ways multiple marginalized and oppressed social statuses play a role in vulnerability to adverse health, social, and economic outcomes. Frameworks such as the Matrix of Domination (Collins, 1986) and Intersectionality (Crenshaw, 1989), which draw from Black feminist traditions (e.g., Combahee River Collective, 1995), aim to explain the compounding effects of multiple forms of discrimination and prejudice on Black women. These frameworks have since been used to help to frame other forms of intersectional oppression and health disparities (see, e.g., Bowleg, 2012).

Research has demonstrated strong associations between structural oppression and stigmatization (or identity with a group that experiences these) and economic instability across many populations. Due to racist policies and systems in the United States, racialized minority status is a strong and pervasive predictor of poverty as well as many other indicators of reductions in economic well-being (Baker et al., 2022; Williams & Collins, 2001). Other factors such as having a disability (Wang, 2005), being younger (Shrider et al., 2021), and not having a college education (Aikens & Barbarin, 2008; Orr, 2003) have also been found to predict lower incomes and poverty across the U.S. population. Research has indicated that many of these factors play a role in poverty and economic outcomes among sexual minority groups, specifically (Wilson et al., 2021). For example, racialized minority same-sex couples and individuals, especially Black couples and people, have among the lowest incomes compared with their white counterparts (Badgett et al., 2021; Kastanis & Wilson, 2014; Wilson et al., 2021). Black feminist conceptual frameworks have been used in prior research to understand the role of sexual orientation, gender expression and identity, and racialization in producing and/or compounding these types of documented economic disparities (Wilson, Gomez, et al., 2020). This study draws on this set of frameworks to help conceptualize the potential measured and unmeasured factors associated with sexual minority women’s health and economic insecurity in terms of their experiences as both sexual minorities and women, as well as at the intersection of race, age, and other social statuses.

## Methods

### Study Design

Respondents participated in the Generations survey, a 5-year longitudinal study designed to assess health and social experiences across three generations of LGB people (Meyer et al., 2016). The survey research consulting company, Gallup, recruited participants using the Gallup Daily Tracking Survey (GDTS) between 2016 and 2017. Preliminary eligibility was determined using a single question on the GDTS: “Do you personally identify as lesbian, gay, bisexual, or transgender?” Those responding affirmatively were further assessed for final eligibility. Respondents were eligible if, at recruitment, they identified as lesbian, gay, bisexual, queer, or same-gender loving; were not transgender; and were in one of three age cohorts: 18–25, 34–41, and 52–59 years. These age cohorts represent distinct historical contexts relevant to the social life of sexual minorities in the United States (Krueger et al., 2020). The investigators developed the framework for these cohorts by composing a list of major events relevant to the social environment of LGB people starting in 1969 (further details on which events were included are available on the study website at www.generations-study.com). The Daily Tracking survey is a daily (350 days per year) telephone interview of a national probability sample of 1,000 adults ages 18 and older, inquiring about topics that included the respondents’ politics, economics, and general well-being. Gallup respondents include English- and Spanish-speaking individuals from all 50 U.S. states and the District of Columbia. Though not all adult age groups in the U.S. population are included, the sample is representative of the population of people in the targeted age cohorts in the United States.

Eligible participants identified as white, Black, or Latinx; completed a sixth-grade education or higher; and spoke English. Interested eligible respondents then provided oral consent and were provided access to the baseline Generations survey, either online or by mail. Respondents were compensated with $25 gift certificates or cash at each wave. Follow-up waves were conducted in 2017 and 2018. Data from Wave 1 were used for these analyses. All analyses (bivariate and multivariate) were restricted to lesbian/gay and bisexual identified women (respondents whose sex at birth was female and who identified as a woman at study onset [N = 679]), and multivariate analyses were additionally restricted to respondents with valid responses for psychological distress, partnership status, and covariates. The Generations study was reviewed and approved by the University of California, Los Angeles Office of the Human Research Protection Program.

### Study Variables

#### Sexual Identity and Gender

Respondents in the Generations study were asked, “Which of the following best describes your current sexual orientation?” Those who identified as women in the eligibility screening and whose sexual identity was lesbian, gay, or same-gender loving were categorized as “lesbian women.” Those who identified as women in the eligibility screening and whose sexual identity was bisexual were categorized as “bisexual women.”

#### Poverty

The poverty level is very low, so for this study we doubled it to 200% of the federal poverty level (FPL), as is common in research and policy analysis. For example, when the survey was administered in 2017, a single person household with a household income that is less than or equal to approximately $25,500 per year has an income that is 200% of the federal poverty level (U.S. Census Bureau, 2023). For a family of four (including two children), 200% of the FPL is a household income of $49,700 or less per year.

#### Age

Respondents were prompted with “Please tell me your age.” Those who were between the ages of 18 and 25, 34 and 41, and 52 and 59 years were eligible for the Generations study and were included in this analysis.

#### Race/Ethnicity

In the Generations study, Black and Latinx respondents were oversampled to increase their representation in the sample; however, American Indian/Alaska Native and Asian/Asian American/Pacific Islander respondents were not recruited (except those who were multi-racial and also identified as white, Black, or Latinx) because estimates of recruitment showed that the researchers could not recruit a sufficient number in the target age groups to allow for meaningful statistical analyses. For the current study, respondents who identified as Latinx, regardless of any additional racial or ethnic identities chosen, were categorized as Latinx. Respondents who identified as Black and not Latinx, regardless of any additional races chosen, were categorized as Black. Last, respondents who identified as white and did not identify as Latinx or Black were categorized as white.

#### Education

Respondents were asked, “What is the highest level of school you have completed or the highest degree you have received?” Responses were dichotomized as “high school degree or less” and “attended some college or more.”

#### Children

Respondents were asked, “Do you have any children?” followed by a question asking whether the children were younger than or older than 18 and whether or not they lived in the home of the respondent. For the current study, we created three groups for the analyses: 1) respondents without children; 2) respondents with children younger than 18 living in the household; and 3) a combined group including those with children younger than 18 not living in the household and those who are parents to adult children.

#### Partner Gender

Respondents were asked, “Are you currently in a relationship or feel a special commitment to someone?” Those who selected “no” were categorized as having “no partner.” Those who selected “yes” were asked, “What is your current partner’s gender?” Given the study’s goal to test predictors of poverty, and because cisgender men are known to have higher incomes on average than all other gender groups (Badgett et al., 2019, 2021), we created two categories of relationships: current relationship with a cisgender man and not currently in a relationship with a cisgender man. Respondents who selected “man, non-transgender” were labeled “cisgender man” (*n* = 255), and those who selected “woman, non-transgender,” “transgender,” or “nonbinary/genderqueer” for their partner’s gender were labeled “woman or TNB [transgender/nonbinary]” (*n* = 278).

#### Gender Expression

Respondents were asked, “A person’s appearance, style, or dress may affect the way people think of them. On average, how do you think people would describe your appearance, style, or dress?” Response options ranged from 1 (very feminine) to 7 (very masculine), with a middle option (4) of “equally feminine and masculine.”

#### Out to Coworkers

Respondents reported to what degree they were “out” about their sexual orientation to coworkers on a scale ranging from 1 to 5. Responses were categorized as those who selected “none” versus those who selected “some, most, or all” versus “don’t know/does not apply.” Single-item measures of outness have proven to be as strong as multi-item indicators of outness (Wilkerson et al., 2016).

#### Felt Stigma

Expectations of stigmatization were measured using the 3-item Felt Stigma scale (Herek, 2008). Respondents indicated their agreement on a 5-point Likert scale with items such as “most employers where I live will hire openly LGB people if they are qualified for the job” and “most people where I live would not want someone who is openly LGB to take care of their children.” Values are a mean calculated from the mean score of each item on the scale. Higher values represent greater felt stigma (α = .68).

#### Everyday Discrimination

From a list of nine, respondents reported the frequency with which they had experienced common incidences of discrimination in the past year (Williams et al., 1997). Experiences included “being treated with less courtesy than other people” and “being called names or insulted.” Answer options ranged from “never” to “often” on a 4-point Likert scale. Values are a mean calculated from the mean score of each item on the scale. Higher values represent greater frequency of discriminatory experiences (α = .91).

#### Internalized Homophobia

Using the 5-item Internalized Homophobia Scale (Herek et al., 2009), respondents indicated their agreement on a 5-point Likert scale with items such as “I have tried to stop being attracted to people who are the same sex as me” and “I feel that being LGB is a personal shortcoming for me.” Values are a mean calculated from the mean score of each item on the scale. Higher values represent greater internalized homophobia (α = .74). Though a measure for biphobia was available in this dataset in Wave 2 for bisexual identified respondents, the measure was not correlated with poverty and inclusion of a variable from Wave 2 would have greatly diminished the sample size; therefore, it was not included in the models.

#### Psychological Distress

Using the 6-item Kessler-6 Distress Scale (Kessler et al., 2003), respondents indicated on a 5-point Likert scale how often in the past 30 days they felt symptoms such as nervousness or hopelessness (α = .90).

#### Analysis

Table 1 displays weighted estimates of population characteristics with associated 95% confidence intervals for lesbian and bisexual women; these are calculated from observations that had non-missing sexual identity among women.

Associations of risk factors with the outcome poverty were estimated in logistic regression models. To avoid deleting observations with missing values and biased estimation resulting from deletion, we multiply imputed missing values by chained equations, resulting in 20 imputed data sets. Psychological distress (*n* = 27), felt stigma (*n* = 7), everyday discrimination (*n* = 20), internalized homophobia (*n* = 14), and urbanicity (*n* = 22) were imputed via predictive mean matching; poverty (*n* = 10) was imputed via logistic regression; and partner gender identity (*n* = 8), children (*n* = 23), outness at work (*n* = 8), and sexual identity: lesbian/gay, bisexual, queer/pansexual (*n* = 31) were imputed via multinomial logistic regression. Variables that were not imputed but included in the both the imputation and analysis models were: age, sex at birth, race/ethnicity, education, region, and the full sample weight.

Bivariate associations expressed as odds ratios that relate risk factors to poverty are shown in Table 2, separately for lesbian and bisexual women, and were estimated in logistic regression models where poverty was regressed on a single risk factor, sexual identity (lesbian vs. bisexual), and their interaction, to assess if the risk factor associations differed between the two groups. The resulting odds ratios thus represent unadjusted relationships between the risk factors and poverty, within each sexual identity. Adjusted odds ratios (AORs) were then estimated from a logistic regression model including all risk factors, sexual identity, and the interaction of each risk factor with sexual identity; these are shown in Table 3 and discussed below, separately for lesbian and bisexual women. Table 3 also displays interaction coefficients expressing the factor change comparing AORs for lesbian and bisexual women. Confidence intervals and *p*-values for null hypothesis tests accompany all reported odds ratios and interaction coefficients. Risk factors not discussed in the following sections had very small effect sizes or were estimated with too much uncertainty to make clear inferences. All logistic regressions were weighted with a nationally representative sampling weight.

## Results

Table 1 shows that bisexual women were considerably more likely to be in poverty (53.68%) than lesbians (40.82%). Lesbians were on average older (M = 34.53) and more variable (SD = 15.74) in age than bisexual women (M = 26.62, SD = 8.63). Latinx/Hispanic representation was similar across lesbian and bisexual women; however, bisexual women were slightly more likely to identify as white and lesbians slightly more likely to identify as Black. Lesbians were more likely to have education beyond high school (61.15%) than bisexual women (52.40%). Approximately one-third of both groups of women reported being single, and bisexual women were much more likely to have a cisgender man (58.67%) than a woman or TNB person (7.67%) as a partner. Approximately one-quarter of both groups of women had children; however, bisexual women were more likely to have children younger than 18 in the household (20.27% vs. 12.72% for lesbians). Lesbians (78.41%) were much more likely to be out to their coworkers than bisexual women (44.94%) and were more likely to have masculine than feminine gender presentation. Although the two groups of women reported similar levels of felt stigma and internalized homophobia, bisexual women reported higher levels of everyday discrimination and psychological distress.

### Lesbian Women

Table 2 shows the bivariate relationship between the population characteristics and poverty rate among lesbians and bisexual women. Age was associated with poverty among lesbian women, with poverty being slightly less likely as age increased (OR [95% confidence interval (CI)]: .97 [.95, .99]). Black and Latinx lesbian women had significantly higher odds of experiencing poverty compared with white lesbian women (OR [95% CI]: 5.09 [2.36, 10.96] and 2.61 [1.17, 5.79], respectively). Having a college education was related to lower odds of being in poverty for lesbian women by 70% (OR [95% CI]: .30 [.15, .59]). Respondents reporting a masculine gender expression had an approximately 40% higher odds of poverty than those who were not masculine-presenting among lesbian women (OR [95% CI]: 1.39 [1.12, 1.71]). Additionally, higher scores of stigma and discrimination were associated with higher odds of being in poverty by almost 85% (OR [95% CI]: 1.84 [1.28, 2.63] and 1.84 [1.17, 2.90], respectively). Experiencing symptoms of psychological distress was associated with a higher odds of being in poverty for lesbian women (OR [95% CI]: 1.16 [1.09, 1.24]). Partnership status, partner’s gender, having children, outness, and internalized homophobia were not significantly associated with poverty among lesbian women.

With regard to the tested models of the combined set of predictors for lesbians, Table 3 shows that compared with white lesbians, Black lesbians had more than four times the odds of poverty (AOR = 4.38, 95% CI [1.61, 11.89]), while Latinx/Hispanic lesbians had three times the odds (AOR = 3.25, 95% CI [1.26, 8.40], respectively). Lesbians who were educated beyond high school had one-third of the odds of poverty of those who were not (AOR = .36, 95% CI [.16, .082]). Higher levels of masculine presentation (AOR = 1.42, 95% CI [1.11, 1.81]), felt stigma (AOR = 1.83, 95% CI [1.08, 3.09]), and psychological distress (AOR = 1.16, 95% CI [1.07, 1.25]) were associated with higher odds of poverty among lesbians. Experiencing discrimination was the only factor whose association with poverty was no longer significant after adjusting for the other characteristics. After adjusting for other factors, we found that having a child younger than 18 living at home was associated with higher odds of experiencing poverty among lesbian women (AOR = 3.18, 95% CI [.95, 10.68]).

### Bisexual Women

The bivariate analysis of the relationship between population characteristics and poverty rate among bisexual women found that Black bisexual women had more than two times higher odds of experiencing poverty compared with white bisexual women (OR [95% CI]: 2.37 [1.20, 4.71]). Those with a college education had lower odds of being in poverty than those without a college education among bisexual women by 38% (OR [95% CI]: .62 [.37, 1.02]). Bisexual women with children, both younger than 18 living in the household and adult children, had approximately two and a half to more than three times higher odds of being in poverty than bisexual women who had never had children (OR [95% CI]: 3.47 [1.87, 6.45] and 2.47 [1.07, 5.67], respectively). Experiencing discrimination was associated with higher likelihood of being in poverty by almost 50% among bisexual women (OR [95% CI]: 1.49 [1.04, 2.14]). Experiencing symptoms of psychological distress was associated with slightly higher odds of being in poverty for bisexual women (OR [95% CI]: 1.05 [1.00, 1.10]). Age, being Latinx/Hispanic compared with white, partnership status, partner’s gender, gender expression, outness, stigma experiences, and internalized homophobia were not significantly associated with poverty among bisexual women.

Table 3 shows the tested models of the combined set of predictors for bisexual women. Though the racial differences were not as pronounced for bisexual women as they were for lesbians, Black bisexual women still had higher odds of poverty compared to white bisexual women (AOR = 2.75, 95% CI [1.34, 5.66]); however, Latinx/Hispanic bisexual women were not significantly different from white bisexual women, though their effect size was larger (AOR = 1.62, 95% CI [.80, 3.27], respectively). Being educated beyond high school was again associated with lower odds of poverty, although less so for bisexual women (AOR = .61, 95% CI [.34, 1.10]) than lesbians. Having children of any age was strongly associated with poverty for bisexual women, with a greater than five times higher odds (AOR = 5.66, 95% CI [2.79, 11.50] for bisexual women with children younger than 18 in the household and 6.46 [2.06, 20.20] for bisexual women with adult children). As with lesbian women, for bisexual women the association between experiencing more frequent discrimination and poverty was no longer significant after adjusting for the other characteristics. Unlike lesbian women, reported psychological distress (AOR = 1.04, 95% CI [.98, 1.09]) was no longer associated with the likelihood of experiencing poverty once the other factors were entered into the model.

### Differences Between Model Coefficients

With regard to whether the adjusted associations between model predictors and poverty differed between lesbians and bisexual cisgender women, exponentiated interaction coefficients expressing the factor change between the AORs of the two subgroups indicate that the differences are not large or were estimated with too much uncertainty to permit clear inference for most of the AORs (see Table 3). However, the data provided clearer evidence of different AORs for gender expression (Ratio AOR = .71, 95% CI [.51, .99]) and psychological distress (Ratio AOR = .90, 95% CI [.82, .98]), indicating that compared with lesbian-identified women, bisexual identified women showed a smaller positive association between masculine gender expression and poverty and a smaller positive association between poverty and psychological distress, respectively.

## Discussion

As expected, given previous research, bisexual women were significantly more likely to report living with poverty level incomes, and they also differed from lesbian women on many of the expected psychological and social characteristics. Bisexual women in this sample were younger, were more likely to be partnered with cisgender men (if partnered), were less educated, reported higher levels of psychological distress, and were less likely to be out as a sexual minority to their coworkers compared with lesbian women. The primary aim of this study was to understand whether this constellation of demographic, psychological, and social factors predicted poverty among bisexual and lesbian women similarly. Dimensions of race (i.e., identifying as Black) and education (i.e., having a high school diploma or less) were associated with living in poverty for both groups, although they were more salient among lesbians. Yet, our study indicated that some factors were differentially important for predicting poverty among bisexual women and lesbian women.

With regard to the significance of race and education in models predicting poverty among lesbian and bisexual cisgender women, these findings are unsurprising. There is a strong body of research that has documented the interlocking relationships among race, education, and poverty across the U.S. population, indicating that these factors represent a complex system of structures that are associated in a multidirectional way. Racialized minority groups, such as Black/African American, Latinx, and American Indian people have persistently reported lower average incomes when compared with white/European Americans in the United States, and poverty itself is often understood as a function of racist policies and structures (Beech et al., 2021; Brown, 2022). Lower education levels have been found to operate as both an effect of poverty and an independent predictor (Aikens & Barbarin, 2008; Orr, 2003). As such, finding that race and education are associated with poverty among lesbians and bisexual cisgender women is not novel, as there were no reasons to assume women who are sexual minorities would not also experience the effects of race and socioeconomic status. Research on sexual minorities has also indicated higher rates of economic disparities at the intersection of race, gender, and sexual orientation, particularly in comparison with white cisgender straight people and sexual minorities (Wilson et al., 2022). Yet, it is notable that some of the other factors tested in this study remain significantly associated with poverty with these well-established covariates included in the model.

The role of minority stressors such as outness, everyday discrimination, and internalized homophobia did not significantly predict poverty for either group. However, reporting experienced stigma related to one’s sexual orientation was associated with living in poverty among lesbians but not bisexual women. Previous research has indicated that although bisexual women report higher levels of overall everyday discrimination, they are less likely to attribute that to their sexual orientation (Wilson et al., 2021). Further, it appears that even among bisexual women who report experiencing this stigma, it may be in settings, such as at home or in LGBT community settings, in which there is a less direct relationship to economic instability. For example, research has demonstrated that lesbians report experiencing discrimination in employment settings or in public, often as a function of prejudice against nonconforming gender expression (Dozier, 2017; Levitt et al., 2012). Yet, research has not indicated this is a salient experience for bisexual women specifically. It is possible that the setting in which discrimination and stigma is experienced, and the social status targeted, may differentially affect economic outcomes among LGBT subgroups. That is, if lesbians are more likely to experience stigma specific to their sexual orientation in work or other public settings specifically (in part also due to higher levels of gender nonconformity and masculine presentation or rejection of expectations to partner with men), that could explain why this factor was relevant for poverty (perhaps via reduced experiences in employment opportunities) among lesbian women and not bisexual women, even if both groups experience stigma overall.

Surprisingly, psychological distress was not associated with poverty for bisexual women, but it was for lesbians. This was unexpected given previous research establishing a strong association between mental health and poverty overall, and the high rates of psychological distress among bisexual women. It is possible that psychological distress was a significant predictor of poverty for lesbians but not for bisexual women because there is a differential relationship between psychological distress and the other factors in the models for the two groups. The impact of whether respondents had children on poverty may provide an example of this complexity. Although similar proportions of bisexual and lesbian women reported having children in their lifetime, bisexual women were more likely to be living in a household with children younger than 18 years. The characteristics and experiences of parenting may differ in ways that impact how the full set of factors (having children, psychological distress, racialized experiences) operate together to predict poverty. For example, prior research has also indicated that bisexual women are more likely to have children living in the home (i.e., they are parents of younger children), which can present significant economic challenges (Pew Research Center, 2013; Wilson & Bouton, 2024). In addition, research has shown that parenthood is also independently associated with psychological distress and other mental health concerns (Assink et al., 2022; Tordoff et al., 2024). It is possible that the predictive power of psychological distress alone on poverty is diminished by the effect of the overarching stress and economic challenges associated with raising and providing for children, an experience more common among bisexual women than among lesbians. This is one plausible explanation of the ways mental health, parenting status, and poverty may interact differently among these two sexual minority subgroups of cisgender women. Additional research is needed to further understand the mechanisms behind these findings.

This is the first study to assess possible explanatory factors for subgroup differences in economic well-being among bisexual and lesbian cisgender women using population-based data. The findings indicate that the differences in proportions of those experiencing poverty among cisgender bisexual women compared with lesbian women may be a function of both the strength and prevalence of psychological and contextual factors among the subgroups. Nonetheless, the study has limitations that must be considered. This study relied on data collected as part of a generational cohort study in which certain age groups were intentionally excluded. It is possible that there is a relationship between some of the factors studied and age that may impact the results of the analyses. For example, if experienced stigma or having children is more or less likely in the 26- to 33-year-old group that was excluded from the sample, this may have resulted in an error of estimation of the association between these factors and poverty. As more population-based data inclusive of measures of sexual orientation identity along with economic and minority stress measures become available, it will be useful to assess whether the findings hold across a continuous age group sample. Another feature of the sample that limits the conclusions across racialized groups is that it excluded people whose identity was Asian, American Indian, or Alaska Native. The exclusion was due to the funding and time to recruit enough LGB respondents in these groups and has been discussed in previous literature (Meyer et al., 2020).

In addition to these sampling issues, there are limitations to using cross-sectional data in efforts to study predictors of economic instability that must be considered as context for these findings. Although the study framework used existing research to conceptually model poverty as an outcome to the tested factors, the direction of the relationships between some factors such as having children, discrimination and stigma associated with sexual orientation and gender expression, and mental health concerns could reasonably be explained in a differently hypothesized directional relationship to poverty. This study established that these factors are strongly associated with poverty, and differently so among these two subpopulations of sexual minority cisgender women, but future research should consider examining longitudinal data that allows for a more nuanced assessment of the ways and at what time points experiences as a sexual minority, shifts between genders of partners over time, experiences with parenthood, and other factors affect (or are affected by) poverty rates.

### Implications for Policy and/or Practice

The findings of this study have implications for how pathways in and out of poverty may look similar or different for lesbian and bisexual cisgender women. The role of racialized status, education, parenting, and stigma indicate some shared and unique points of intervention to address economic well-being. Future research that explicates the timeline of experienced stigma and parenting in relationship to poverty would help further our understanding of how these findings map on to lived experience and the impact of structural and systemic factors. In addition, qualitative research that highlights the nuances of how these psychological and contextual factors play a role in the day-to-day lives of queer women will further our understanding of their relevance in economic well-being and how they might contribute to economic disparities between sexual minority groups and in comparison with sexual majority groups (especially as compared with heterosexual cisgender women). Given the findings of this study, future lines of inquiry that delineate factors specific to bisexual (e.g., biphobia) or lesbian (e.g., antilesbian bias) women related to poverty and other economic outcomes would be helpful in making sense of these observed differences. Understanding these mechanisms is key to effective health and economic policy development for LGBT people. Advocates of key legislation, such as the Equality Act, or supporters of the U.S. Supreme Court’s decision in *Bostock v. Clayton County* tend to highlight the significant relationship between discrimination in the workplace and elsewhere as factors that produce LGBT economic disparities (see, e.g., Santos et al., 2021). Our findings indicate that this mechanism (i.e., sexual orientation–related discrimination) may be a driver of low incomes for some LGBT subgroups, such as lesbians, but not others. The findings also point to the potential positive impact of parenting-related policy interventions, such as the Earned Income Tax Credit (EITC), for bisexual women in particular as a pathway out of poverty. In essence, this study contributes to the framing of policy solutions to LGBT poverty in ways that acknowledge the diversity of experiences and social factors among subgroups.

## Figures and Tables

**Table 1:** Weighted Estimates of Population Characteristics

Variable	Categories	Lesbian Women (n = 324)	Bisexual Women (n = 355)
		Mean (SD) or Freq % [95% CI]	Mean (SD) or Freq % [95% CI]
Poverty	Income < 200% FPL	40.82% [33.89%, 48.15%]	53.68% [47.38%, 59.87%]
Age	Range: 18–59	34.53 (15.74) [32.64, 36.42]	26.62 (8.63) [25.57, 27.66]
Race/ethnicity	White	56.55% [49.44%, 63.41%]	64.72% [58.71%, 70.29%]
	Black	23.73% [18.12%, 30.42%]	16.45% [12.55%, 21.28%]
	Latinx/Hispanic	19.72% [14.59%, 26.11%]	18.83% [14.58%, 23.97%]
Education	High school or less	38.85% [31.61%, 46.62%]	47.6% [41.28%, 53.99%]
	Some college or more	61.15% [53.38%, 68.39%]	52.40% [46.01%, 58.72%]
Partner gender	Woman or TNB	65.66% [58.71%, 71.99%]	7.67% [5.21%, 11.15%]
	Cisgender man	0.18% [0.03%, 1.30%]	58.67% [52.36%, 64.72%]
	No partner	34.16% [27.82%, 41.11%]	33.66% [27.91%, 39.94%]
Children	None	77.02% [71.00%, 82.10%]	73.51% [67.89%, 78.45%]
	Younger than 18 and in the household	12.72% [8.71%, 18.21%]	20.27% [15.80%, 25.62%]
	Adult children or children younger than 18 not living in the household	10.26% [7.31%, 14.22%]	6.22% [4.16%, 9.20%]
Gender expression	Range: Very feminine (1)–very masculine (7)	3.80 (1.89) [3.53, 4.07]	2.91 (1.06) [2.76, 3.06]
Out to coworkers	No	6.39% [3.73%, 10.75%]	30.49% [25.11%, 36.46%]
	Yes	78.41% [71.93%, 83.73%]	44.94% [38.80%, 51.23%]
	Don’t know/not applicable	15.20% [10.67%, 21.19%]	24.57% [19.35%, 30.67%]
Felt stigma	Range: 1–5	2.75 (.97) [2.62, 2.88]	2.76 (.77) [2.65, 2.87]
Everyday discrimination	Range: 1–4	1.94 (.75) [1.84, 2.05]	2.2 (.64) [2.11, 2.30]
Internalized homophobia	Range: 1–5	1.61 (.79) [1.50, 1.72]	1.61 (.60) [1.52, 1.69]
Psychological distress	Range: 0–24	7.27 (5.65) [6.51, 8.03]	10.64 (4.79) [9.95, 11.33]

*Abbreviations:* FPL, federal poverty level; TNB, transgender/nonbinary.

Generations Study, Wave 1 (2016–2017).

**Table 2:** Bivariate Odds Ratio Estimates Associating Variables With Poverty

Variable	Lesbian Women	Bisexual Women
	OR [95% CI]	OR [95% CI]
Age	**.97 [.95, .99]**	1.00 [.98, 1.02]
Black identity (vs. white)	**5.09 [2.36, 10.96]**	**2.37 [1.20, 4.71]**
Latinx/Hispanic identity (vs. white)	**2.61 [1.17, 5.79]**	1.27 [.68, 2.38]
More than high school (vs. high school or less)	**.30 [.15, .59]**	**.62 [.37, 1.02]**
No partner (vs. has partner)	1.32 [.71, 2.44]	1.30 [.53, 3.19]
Cis male partner (vs. cis woman/TNB partner)	2.12 [.58, 7.74]	1.61 [.68, 3.80]
Children younger than 18 in HH (vs. no children)	1.43 [.61, 3.34]	**3.47 [1.87, 6.45]**
Children any age not in HH (vs. no children)	.62 [.26, 1.46]	**2.47 [1.07, 5.67]**
Gender expression	**1.39 [1.12, 1.71]**	1.0 [.90, 1.34]
Out to coworkers (vs. not out)	.95 [.29, 3.13]	1.01 [.57, 1.77]
Don’t know if out/NA (vs. not out)	1.95 [.49, 7.73]	.70 [.35, 1.37]
Felt stigma	**1.84 [1.28, 2.63]**	1.26 [.95, 1.66]
Everyday discrimination	**1.84 [1.17, 2.90]**	**1.49 [1.04, 2.14]**
Internalized homophobia	1.00 [.67, 1.48]	1.01 [.70, 1.45]
Psychological distress	**1.16 [1.09, 1.24]**	**1.05 [1.00, 1.10]**

*Abbreviations:* CI, confidence interval; Cis, cisgender; HH, household; NA, not applicable; OR, odds ratio; TNB, transgender/nonbinary.

Note: Bolded values indicate statistical significance for *p*-values < .05 and pronounced effect sizes.

Generations Study, Wave 1 (2016–2017).

**Table 3:** Adjusted Odds Ratio Estimates Associating Variables With Poverty

Variable	Lesbian Women		Bisexual Women		Comparison*	
	OR [95% CI]	*p*	OR [95% CI]	*p*	OR [95% CI]	*p*
Age	1.00 [.97, 1.03]	.837	.97 [.94, 1.01]	.105	.98 [.93, 1.02]	.307
Black identity (vs. white)	**4.38 [1.61, 11.89]**	**.004**	**2.75 [1.34, 5.66]**	**.006**	.63 [.18, 2.17]	.462
Latinx/Hispanic identity (vs. White)	**3.25 [1.26, 8.40]**	**.015**	1.62 [.80, 3.27]	.176	.50 [.15, 1.65]	.253
More than high school (vs. high school or less)	**.36 [.16, .82]**	**.015**	**.61 [.34, 1.10]**	.101	1.70 [.61, 4.76]	.313
No partner (vs. has partner)	1.44 [.66, 3.16]	.363	1.44 [.48, 4.37]	.519	1.00 [.25, 3.96]	1.00
Cisgender male partner (vs. cisgender woman/TNB partner)	1.41 [.20, 10.14]	.732	1.38 [.47, 4.07]	.965	.98 [.10, 9.30]	.987
Children <18 years (vs. no children) in HH	**3.18 [.95, 10.68]**	.061	**5.66 [2.79, 11.50]**	**<.001**	1.78 [.44, 7.24]	.420
Children any age not in HH (vs. no children)	1.51 [.50, 4.54]	.464	**6.46 [2.06, 20.20]**	**.001**	4.28 [.88, 20.90]	.072
Gender expression	**1.42 [1.11, 1.81]**	**.005**	1.01 [.80, 1.27]	.965	**.71 [.51, .99]**	**.044**
Out to coworkers (vs. not out)	.60 [.10, 3.39]	.558	.93 [.48, 1.79]	.823	1.56 [.24, 10.27]	.644
Don’t know if out/NA (vs. not out)	1.25 [.19, 8.07]	.811	**.45 [.21, .95]**	.037	.36 [.05, 2.77]	.322
Felt stigma	**1.83 [1.08, 3.09]**	**.025**	1.22 [.90, 1.66]	.206	.67 [.36, 1.23]	.196
Everyday discrimination	.71 [.35, 1.46]	.350	1.12 [.73, 1.73]	.605	1.58 [.68, 3.65]	.286
Internalized homophobia	.80 [.48, 1.33]	.381	.92 [.62, 1.35]	.672	1.16 [.61, 2.21]	.658
Psychological distress	**1.16 [1.07, 1.25]**	**<.001**	1.04 [.98, 1.09]	.217	**.90 [.82, .98]**	**.022**

*Abbreviations:* AOR, adjusted odds ratio; CI, confidence interval; HH, household; NA, not applicable; TNB, transgender/nonbinary.

Note: Bolded values indicate statistical significance for *p*-values < .05 and pronounced effect sizes.

* Exponentiated interaction coefficient expressing the factor change between the AORs of lesbians and bisexual women and *p*-value for hypothesis test of this change against null value of 1.

Generations Study, Wave 1 (2016–2017).
